# Supplementary material for: Purkinje cell number-correlated cerebrocerebellar circuit anomaly in the valproate model of autism
Source: Sci Rep. 2019 Jun 25;9:9225. doi: 10.1038/s41598-019-45667-1 (PMC6592903; doi:10.1038/s41598-019-45667-1)
Supplement: Supplementary file 1 — Supplementary Material [file 41598_2019_45667_MOESM1_ESM.docx]

Supplementary Material

For the manuscript entitled:

## Purkinje cell number-correlated cerebrocerebellar circuit anomaly in the valproate model of autism

Tamás Spisák^1,2#^, Viktor Román^1#^, Edit Papp^3^, Rita Kedves^1^, Katalin Sághy^1^, Cecília Katalin Csölle^1^, Anita Varga^1^, Dávid Gajári^1^, Gabriella Éva Nyitrai^1^, Zsófia Spisák^1^, Zsigmond Tamás Kincses^1,4^, György Lévay^1^, Balázs Lendvai^1^, András Czurkó^1*^

* Correspondence to [czurkoa@richter.hu](mailto:czurkoa@richter.hu)

^#^ Both authors contributed equally to this work.

^1^ Pharmacology and Drug Safety Research, Gedeon Richter Plc., Budapest, Hungary;

^2^ Department of Neurology, University Hospital Essen, Essen, Germany;
^3^ Institute of Experimental Medicine, Hungarian Academy of Sciences, Budapest, Hungary;
^4^ Department of Neurology, University of Szeged, Szeged, Hungary

# Supplementary Tables

*Supplementary Table 1. Summary statistics of the weight, average in-scanner motion (mean RMS of relative displacement), mean isoflurane dose and mean respiration rate of the rats involved in experiment (N=36).*

|  | VEHICLE | | | VPA400 | | | VPA600 | | |
| --- | --- | --- | --- | --- | --- | --- | --- | --- | --- |
| MRI 1 | Mean(±sd) | min | max | Mean(±sd) | min | max | Mean(±sd) | min | max |
| Weight (g) | 196.0(±27.1) | 164 | 230 | 196.3(±30.4) | 154 | 243 | 210.4(±25.1) | 171 | 246 |
| Average motion (mm) | 0.01(±0.006) | 0.003 | 0.03 | 0.006(±0.003) | 0.002 | 0.013 | 0.006(±0.003) | 0.002 | 0.012 |
| Isoflurane dose (%) | 1.47(±0.18) | 1.1 | 1.7 | 1.52(±0.14) | 1.3 | 1.7 | 1.47(±0.15) | 1.3 | 1.7 |
| Respiration rate (1/min) | 64.4(±4.4) | 55 | 71 | 66.4(±2.9) | 61 | 71 | 64.2(±5.3) | 56 | 71 |
| MRI 2 |  |  |  |  |  |  |  |  |  |
| Weight (g) | 385.4(±16.6) | 356 | 416 | 391.6(±29.4) | 350 | 435 | 401.0(±20.4) | 371 | 443 |
| Average motion (mm) | 0.005(±0.002) | 0.002 | 0.01 | 0.003(±0.002) | 0.002 | 0.006 | 0.006(±0.003) | 0.003 | 0.013 |
| Isoflurane dose (%) | 1.83(±0.33) | 1.3 | 2.5 | 1.69(±0.21) | 1.4 | 2.1 | 1.92(±0.28) | 1.5 | 2.5 |
| Respiration rate (1/min) | 67.2(±6.3) | 53 | 76 | 65.8(±5.9) | 58 | 78 | 66(±5) | 58 | 75 |

*Supplementary Table 2. Summary statistics of the gray matter, white matter and whole brain volumes in the VEH, VPA400 and VPA600 group, throughout the two MRI measurements (N=36). Values are given in unit mm^3^ for absolute volumes and as percentage of whole brain volume for relative volumes.*

|  | **VEHICLE** | | **VPA400** | | **VPA600** | |
| --- | --- | --- | --- | --- | --- | --- |
| **MRI1** | Mean | SD | Mean | SD | Mean | SD |
| **Whole brain (mm^3^)** | 2564.8 | 58.5 | 2512.8 | 94.1 | 2483.7 | 37.8 |
| **Gray matter (mm^3^)** | 1619.4 | 44.6 | 1594.4 | 71.1 | 1566.7 | 28.3 |
| **Gray matter (%brain)** | 63.1 | 0.7 | 63.4 | 0.6 | 63.1 | 0.4 |
| **White matter (mm^3^)** | 544.6 | 19.3 | 537.1 | 22.8 | 531.4 | 12.3 |
| **White matter (%brain)** | 21.2 | 0.4 | 21.4 | 0.4 | 21.4 | 0.5 |
| **MR2** |  | | | | | |
| **Whole brain (mm^3^)** | 2646.4 | 57.5 | 2628.8 | 81.7 | 2548.5 | 50.2 |
| **Gray matter (mm^3^)** | 1665.7 | 32.1 | 1648.8 | 56.2 | 1611.2 | 43.6 |
| **Gray matter (%brain)** | 62.9 | 0.9 | 62.7 | 0.9 | 63.2 | 0.9 |
| **White matter (mm^3^)** | 584.0 | 26.6 | 592.0 | 34.8 | 564.3 | 22.4 |
| **White matter (%brain)** | 22.1 | 0.6 | 22.5 | 1.0 | 22.1 | 0.8 |

*Supplementary Table 3. Local maxima of the fMRI BOLD response to somatosensory stimuli in the vehicle group (N=12). Only local maxima above the FWER P>0.05 threshold (Z>4.29) are shown. Minimum peak distance is set to 0.5mm. Peaks are listed by effect size (% BOLD signal change) in decreasing order. Coordinates are given relative to the bregma, according to the atlas of Paxinos and Watson (x: medial-lateral, y: anterior-posterior, z: dorsal-ventral).*

| **x** | **y** | **z** | **% signal change** | **Z-score** | **region** |
| --- | --- | --- | --- | --- | --- |
| -7.1 | -5.4 | -4.8 | 0.46 | 8.37 | Au1 primary auditory cortex (+S2) |
| -4.7 | -3.2 | -2.8 | 0.38 | 10.40 | S1BF primary somatosensory cortex barrel field |
| -5.7 | -6.0 | -2.0 | 0.31 | 7.84 | RSG retrosplenial granular cortex |
| 0.1 | -2.2 | -1.2 | 0.30 | 5.35 | RSA retrosplenial agranular cortex |
| -6.9 | -7.4 | -7.2 | 0.24 | 7.50 | LEnt lateral entorhinal cortex |
| 0.3 | 0.2 | -1.4 | 0.23 | 4.45 | Cg1 cingulate cortex |
| -3.7 | -1.0 | -8.0 | 0.22 | 8.53 | AAD anterior amygdaloid nucleus |
| -6.5 | -9.4 | -5.0 | 0.22 | 5.34 | MEnt medial entorhinal cortex |
| 0.9 | 4.0 | -6.2 | 0.20 | 6.40 | AOM medial anterior olfactory nucleus |
| 4.7 | -3.2 | -6.2 | 0.19 | 6.46 | CPu striatum |
| -6.7 | -2.4 | -7.0 | 0.18 | 5.93 | AIP agranular insular cortex |
| -3.5 | -6.2 | -6.6 | 0.18 | 5.43 | MGV thalamus, medial geniculate nucleus |
| 4.7 | -10.6 | -3.0 | 0.18 | 5.21 | Crus1 cerebellum |
| -3.7 | 0.0 | -3.0 | 0.17 | 4.73 | S1ULp primary somatosensory cortex upper lip |
| -2.9 | 1.4 | -3.2 | 0.17 | 4.84 | S1J primary somatosensory cortex jaw |
| 4.1 | -2.6 | -2.6 | 0.16 | 4.72 | S1BF primary somatosensory cortex barrel field |
| -2.3 | 1.8 | -6.6 | 0.16 | 4.50 | CPu striatum |
| -3.9 | 1.2 | -5.8 | 0.13 | 4.61 | CPu striatum |
| -0.9 | -11.0 | -8.6 | 0.13 | 6.04 | brainstem |
| 2.5 | -0.6 | -7.4 | 0.12 | 5.27 | VP ventral pallidum |
| -2.7 | -10.0 | -7.2 | 0.11 | 4.51 | 1 cerbellum |
| -4.9 | -0.2 | -9.2 | 0.10 | 4.40 | Pir piriform cortex |
| 2.1 | -10.2 | -8.6 | 0.09 | 5.52 | brainstem |
| 3.3 | -11.2 | -2.0 | 0.09 | 4.47 | 6 cerbellum |
| 4.9 | -13.4 | -5.0 | 0.08 | 4.73 | Crus2 cerebellum |
| 5.5 | 1.2 | -7.4 | 0.055 | 4.38 | AIV agranular insular cortex |
| 3.5 | -14.0 | -4.2 | 0.077 | 4.36 | Crus1 cerebellum |

*Supplementary Table 4. Local maxima of the fMRI BOLD response to somatosensory stimuli in the VPA400 group (N=12). Only local maxima above the FWER P>0.05 threshold (Z>4.29) are shown. Minimum peak distance is set to 0.5mm. Peaks are listed by effect size (% BOLD signal change) in decreasing order. Coordinates are given relative to the bregma, according to the atlas of Paxinos and Watson (x: medial-lateral, y: anterior-posterior, z: dorsal-ventral).*

| **x** | **y** | **z** | **% signal change** | **Z-score** | **region** |
| --- | --- | --- | --- | --- | --- |
| -4.9 | -3.0 | -2.8 | 0.68 | 14.30 | S1BF primary somatosensory cortex barrel field |
| -0.3 | -0.8 | -2.4 | 0.48 | 8.47 | Cg2 cingular cortex |
| -0.1 | 3.4 | -5.4 | 0.38 | 11.20 | IL infralimbic and prelimbic cortices |
| -3.9 | -10.0 | -2.8 | 0.36 | 6.11 | 4 cerebellum |
| -7.3 | -6.2 | -7.2 | 0.31 | 8.52 | LEnt lateral entorhinal cortex |
| -3.7 | -0.8 | -8.0 | 0.31 | 10.80 | AAD anterior amygdaloid nucleus |
| 4.1 | -0.6 | -7.8 | 0.28 | 10.10 | CPu striatum |
| -2.5 | 1.6 | -3.0 | 0.27 | 6.11 | S1J primary somatosensory cortex jaw |
| 5.3 | -1.8 | -5.0 | 0.25 | 7.84 | S2 secondary somatosensory cortex |
| 6.1 | -4.8 | -4.4 | 0.25 | 6.88 | Au1 primary auditory cortex |
| -0.5 | -7.2 | -2.6 | 0.24 | 4.74 | SC superior colliculus |
| 4.3 | -10.8 | -3.0 | 0.24 | 5.50 | Crus1 cerebellum |
| 1.9 | 3.0 | -2.6 | 0.21 | 5.51 | FrA frontal association cortex (+M1 motor cortex) |
| -2.1 | -8.4 | -2.8 | 0.19 | 4.36 | RSG retrosplenial granular cortex |
| 2.9 | 2.8 | -5.8 | 0.18 | 7.34 | VO ventral orbital cortex |
| -2.7 | -9.2 | -7.4 | 0.17 | 7.55 | brainstem |
| 2.5 | -0.4 | -2.8 | 0.11 | 4.30 | M1 primary motor cortex |
| -1.9 | -13.6 | -7.2 | 0.11 | 5.66 | 10 cerebellum |
| -2.7 | -14.0 | -4.8 | 0.10 | 5.00 | 8 cerebellum |
| -3.7 | -2.8 | -10.8 | 0.10 | 5.09 | CxA cortex-amygdala transit zone |
| 3.1 | -8.2 | -7.2 | 0.10 | 4.33 | brainstem |
| 2.3 | 2.2 | -8.8 | 0.09 | 5.42 | VP ventral pallidum |
| 4.9 | -13.4 | -6.2 | 0.09 | 5.66 | Crus2 cerebellum |
| 2.9 | -11.8 | -8.2 | 0.09 | 5.07 | brainstem |
| 2.9 | -8.6 | -8.6 | 0.08 | 4.56 | brainstem |
| 2.7 | -14.6 | -3.8 | 0.08 | 4.95 | Crus2 cerebellum |
| 0.9 | -12.6 | -8.6 | 0.07 | 4.51 | brainstem |

*Supplementary Table 5. Local maxima of the fMRI BOLD response to somatosensory stimuli in the VPA600 group (N=12). Only local maxima above the FWER P>0.05 threshold (Z>4.29) are shown. Minimum peak distance is set to 0.5mm. Peaks are listed by effect size (% BOLD signal change) in decreasing order. Coordinates are given relative to the bregma, according to the atlas of Paxinos and Watson (x: medial-lateral, y: anterior-posterior, z: dorsal-ventral).*

| **x** | **y** | **z** | **% signal change** | **Z-score** | **region** |
| --- | --- | --- | --- | --- | --- |
| -6.1 | -4.6 | -3.8 | 0.42 | 8.26 | Au1 primary auditory cortex (+S2) |
| -4.7 | -2.0 | -2.8 | 0.34 | 7.29 | S1BF primary somatosensory cortex barrel field |
| 5.1 | -3.4 | -3.2 | 0.32 | 8.22 | S1BF primary somatosensory cortex barrel field |
| -0.1 | -1.6 | -2.2 | 0.27 | 5.50 | RSGb retrosplenial granular cortex |
| -3.1 | -0.4 | -8.8 | 0.26 | 8.26 | AAD anterior amygdaloid nucleus |
| 1.7 | 1.4 | -2.2 | 0.25 | 6.60 | M1 primary motor cortex |
| -2.3 | -4.6 | -1.6 | 0.24 | 4.99 | PtA parietal association cortex |
| 0.9 | 0.6 | -1.4 | 0.24 | 4.60 | Cg2 cingular cortex |
| 3.1 | -5.0 | -1.6 | 0.23 | 5.16 | CA1 hippocampus |
| -5.3 | -1.8 | -6.0 | 0.22 | 7.75 | CPu striatum |
| -2.5 | 2.4 | -3.0 | 0.21 | 4.31 | S1J primary somatosensory cortex jaw |
| -3.5 | 3.2 | -4.2 | 0.21 | 4.67 | AI agranular insular cortex |
| -6.7 | -7.4 | -7.0 | 0.21 | 6.39 | LEnt lateral entorhinal cortex |
| -1.7 | 3.2 | -6.6 | 0.20 | 6.27 | AOV ventral anterior olfactory nucleus |
| -2.1 | 0.6 | -3.4 | 0.19 | 6.49 | CPu striatum |
| -2.5 | -4.6 | -5.6 | 0.14 | 5.25 | VPM thalamus |
| 3.9 | -0.8 | -7.2 | 0.13 | 4.94 | CPu striatum |
| 2.1 | 2.6 | -5.2 | 0.11 | 4.45 | VO ventral orbital cortex |

*Supplementary Table 6. Group-wise mean(±sd) of the Purkinje cell numbers (count / 100 μm layer length traced) obtained from the cerebellar vermal* *lobules by Calbindin D28k immunostaining.*

|  | VEH | VPA400 | VPA600 |
| --- | --- | --- | --- |
| Lobule 6a | 3.70 (±0.43) | 2.58 (±0.29) | 2.91 (±0.44) |
| Lobule 6b | 2.98 (±0.30) | 2.15 (±0.37) | 2.40 (±0.32) |
| Lobule 6c | 3.15 (±0.43) | 2.23 (±0.36) | 2.32 (±0.36) |
| Lobule 7 | 3.04 (±0.44) | 2.05 (±0.32) | 2.30 (±0.36) |

*Supplementary Table 7. Local maxima of the fMRI BOLD response to somatosensory stimuli in the vehicle group, animals without histological data excluded (N=8). Only local maxima above the FWER P>0.05 threshold (Z>4.22) are shown. Minimum peak distance is set to 0.5mm. Peaks are listed by effect size (% BOLD signal change) in decreasing order.*

| X | Y | Z | Z-score | % signal change | region |
| --- | --- | --- | --- | --- | --- |
| -7.9 | -11.1 | -7.8 | 5.63 | 0.39 | Au1 auditory cortex (+SII) |
| -5.1 | -4 | -2.8 | 7.63 | 0.37 | S1BF primary sensory cortex, barrel filed |
| 0.3 | 3.2 | -2.2 | 4.43 | 0.35 | Cg1 anterior cingulate cortex |
| 0.5 | 3.6 | -5.6 | 6.8 | 0.31 | MO medial orbital cortex |
| -3.9 | -1.0 | -7.6 | 5.26 | 0.16 | Cpu striatum |
| 3.9 | -1.4 | -6.8 | 4.37 | 0.16 | Cpu striatum |
| -6.5 | -8.4 | -7.4 | 5.47 | 0.14 | LEnt entorhinal cortex |
| -6.5 | -4.0 | -9.4 | 5.17 | 0.12 | Pir piriform cortex |
| -4.5 | -5.8 | -8.4 | 4.55 | 0.12 | CA1 hippocampus |

*Supplementary Table 8. Local maxima of the fMRI BOLD response to somatosensory stimuli in the VPA400 group, animals without histological data excluded (N=9). Only local maxima above the FWER P>0.05 threshold (Z>4.22) are shown. Minimum peak distance is set to 0.5mm. Peaks are listed by effect size (% BOLD signal change) in decreasing order.*

| X | Y | Z | Z-score | % signal change | region |
| --- | --- | --- | --- | --- | --- |
| -4. | 9 -3.0 | -2.8 | 19.9 | 0.72 | S1BF primary sensory cortex, barrel filed |
| -6.9 | -4.8 | 4.8 | 8.65 | 0.53 | Au1 auditory cortex (+SII) |
| -0.3 | -0.8 | -2.2 | 6.64 | 0.46 | Cg1 anterior cingulate cortex |
| 0.7 | 4.4 | -5.0 | 10.86 | 0.35 | MO medial orbital cortex |
| -3.5 | -0.6 | -8.0 | 9.52 | 0.35 | Cpu striatum |
| -2.1 | 2.8 | -6.0 | 8.49 | 0.32 | VO ventral orbital cortex |
| -1.9 | -10.2 | -7.6 | 8.43 | 0.27 | brainstem |
| 5.3 | -1.8 | -5.0 | 6.6 | 0.24 | Cpu striatum |
| -3.1 | -12.0 | -5.0 | 4.94 | 0.16 | Crus 2 cerebellum |
| -2.5 | -13.2 | -7.4 | 5.7 | 0.14 | 10 Cerebellum |
| 5.1 | -12.4 | -5.6 | 5.18 | 0.13 | Crus1 Crus2 cerebellum |
| -6.5 | -4.0 | -8.6 | 4.75 | 0.13 | Pir piriform cortex |
| 2.7 | -9.4 | -8.6 | 4.48 | 0.9 | brainstem |
| 2.7 | -14.6 | -3.6 | 4.87 | 0.08 | Crus 2 cerebellum |
| 2.9 | -11.8 | -8.4 | 5.08 | 0.1 | brainstem |

*Supplementary Table 9. Local maxima of the fMRI BOLD response to somatosensory stimuli in the VPA600 group, animals without histological data excluded (N=8). Only local maxima above the FWER P>0.05 threshold (Z>4.22) are shown. Minimum peak distance is set to 0.5mm. Peaks are listed by effect size (% BOLD signal change) in decreasing order.*

| X | Y | Z | Z-score | % signal change | region |
| --- | --- | --- | --- | --- | --- |
| -6.1 | -4.0 | -3.8 | 6.24 | 0.35 | S1BF primary sensory cortex, barrel filed |
| -7.5 | -6.4 | -5.6 | 4.79 | 0.32 | Au1 auditory cortex |
| -2.9 | -0.4 | -9.0 | 6.46 | 0.24 | VP ventral pallidum |
| -1.7 | 3.2 | -6.4 | 6.32 | 0.24 | VO ventral orbital cortex |
| 1.7 | 1.4 | -2.2 | 5.6 | 0.24 | M1 primary motor cortex |
| -6.1 | -8.8 | -6.2 | 6.9 | 0.22 | LEnt lateral entorhinal cortex |
| -1.9 | 0.8 | -3.4 | 4.96 | 0.17 | Cpu striatum |
| 4.1 | -4.0 | -10.0 | 4.72 | 0.17 | PLCo amygdala |
| -6.1 | -5.2 | -8.0 | 5.19 | 0.15 | LEnt lateral entorhinal cortex |
| 3.3 | -0.4 | -8.2 | 4.57 | 0.13 | VP ventral pallidum |
| 2.1 | -9.6 | -8.6 | 4.4 | 0.11 | brainstem |
| -3.9 | 0.6 | -6.2 | 5.93 | 0.20 | Cpu striatum |
| 3.9 | -1.2 | -10.2 | 5.89 | 0.20 | CxA cortex-amygdala transit zone |
| -4.1 | -1.6 | -3.2 | 5.09 | 0.20 | S1 primary somatosensory cortex |

*Supplementary Table 10. Local maxima of the regression analysis of the correlation between the fMRI BOLD response and the normalized Purkinje cell number in the VPA400 group (N=8). Only local maxima above the FWER P>0.05 threshold (Z>4.37) are shown. Minimum peak distance is set to 0.5mm. Peaks are listed by effect size (% BOLD signal change) in decreasing order.*

| X | Y | Z | Z-score | % signal change | region |
| --- | --- | --- | --- | --- | --- |
| 3.3 | 2.8 | -2.2 | 5.64 | 0.69 | M1 primary motor cortex |
| -1.5 | 3.2 | -5.2 | 8.07 | 0.64 | VO ventral orbital cortex |
| -0.1 | 3.4 | -6.6 | 8.98 | 0.62 | DTT dorsal tenia tecta |
| 3.1 | 0.2 | -1.2 | 4.68 | 0.60 | S1FL primary somatosensory cortex forelimb |
| -0.9 | 3.4 | -5.4 | 8.16 | 0.60 | IL infralimbic cortex |
| -0.9 | 3.2 | -4.4 | 7.99 | 0.60 | PrL prelimbic cortex |
| 2.1 | 4.4 | -2.6 | 6.57 | 0.48 | FrA frontal association cortex |
| -7.7 | -4.6 | -6.0 | 5.32 | 0.48 | TeA temporal association cortex |
| 2.7 | -2.0 | -1.6 | 4.51 | 0.48 | S1FL primary somatosensory cortex forelimb |
| -5.5 | -4.2 | -1.8 | 4.95 | 0.4 | S1BF primary somatosensory cortex barrel filed |
| 3.5 | -1.6 | -4.0 | 5.36 | 0.36 | Cpu striatum |
| -3.3 | 0.0 | -9.0 | 5.3 | 0.33 | VP ventral pallidum |
| -4.1 | -11.0 | -7.2 | 6.52 | 0.32 | PFI cerebellum |
| -1.9 | -11.0 | -7.4 | 5.64 | 0.31 | Brainstem |
| -2.5 | -13.4 | -4.0 | 5.95 | 0.30 | Crus2 cerebellum |
| 5.9 | -0.8 | -8.6 | 5.68 | 0.29 | Pir piriform cortex |
| -2.5 | -9.4 | -7.4 | 4.81 | 0.27 | brainstem |
| 4.3 | 2.6 | -6.2 | 4.38 | 0.26 | AI agranular insular cortex |
| -6.1 | -5.6 | -8.6 | 4.79 | 0.25 | LEnt lateral entorhinal cortex |
|  |  |  |  |  |  |

*Supplementary Table 11. Local maxima of the regression analysis of the relationship of the fMRI BOLD response and the normalized Purkinje cell number in the VPA600 group (N=9). Only local maxima above the FWER P>0.05 threshold (Z>4.37) are shown. Minimum peak distance is set to 0.5mm. Peaks are listed by effect size (% BOLD signal change) in decreasing order.*

| X | Y | Z | Z-score | % signal change | region |
| --- | --- | --- | --- | --- | --- |
| -1.3 | 2.8 | -2.0 | -4.78 | -0.91 | M2 secondary motor cortex |
| 0.1 | -6.6 | -1.2 | -5.53 | -0.87 | RSG retrosplenial granular cortex |
| 0.7 | -0.6 | -1.2 | -5.76 | -0.85 | RSA retrosplenial agranular cortex |
| 3.1 | 0.4 | -1.4 | -6.32 | -0.82 | S1FL primary somatosensory cortex forelimb |
| 5.7 | -4.8 | -2.0 | -4.86 | -0.64 | PtA parietal association cortex |
| 4.7 | -4.0 | -1.4 | -5.28 | -0.63 | S1BF primary somatosensory cortex |
| -0.5 | 3.4 | -3.2 | -5.79 | -0.60 | PrL prelimbic cortex |
| -0.7 | -4.6 | -1.4 | -4.81 | -0.58 | RSG retrosplenial granular cortex |
| 3.3 | 1.4 | -1.4 | -5.70 | -0.56 | M1 primary motor cortex |
| 5.3 | -8.6 | -4.4 | -4.49 | -0.38 | PRh perirhinal cortex |
| -6.1 | -3.6 | -8.2 | -5.22 | -0.29 | LEnt lateral entorhinal cortex |
| -2.9 | -12.4 | -5.2 | -4.68 | -0.23 | Crus1-Crus2 cerbellum |
| 2.3 | -10.6 | -5.8 | -4.40 | -0.21 | Lobule 2-3 cerbellum |
| -1.7 | -14.2 | -3.2 | -4.99 | -0.19 | Lobule 6c cerebellum |
| 1.5 | -1.4 | -6.0 | -4.42 | -0.19 | AVVL thalamus |
| -5.3 | -7.6 | -7.8 | -4.58 | -0.18 | LEnt lateral entorhinal cortex |
| -5.3 | -8.2 | -7.8 | -4.69 | -0.16 | LEnt lateral entorhinal cortex |
| -1.3 | -14.0 | -2.4 | -4.70 | -0.14 | Lobule 6b cerebellum |

*Supplementary Table 12. Local maxima of the significant interaction effect of the fMRI BOLD response to somatosensory stimuli between groups and histological data (N=25), where regression slopes between BOLD response and Purkinje cell number are larger in the VPA400 group than that of the vehicle group. Only local maxima above the FWER P>0.05 threshold (Z>4.37) are shown. Minimum peak distance is set to 0.5mm. Peaks are listed by effect size (% BOLD signal change) in decreasing order.*

| X | Y | Z | Z-score | % signal change | region |
| --- | --- | --- | --- | --- | --- |
| -0.9 | 3.0 | -4.2 | 7.45 | 0.83 | PrL prelimbic cortex |
| -1.3 | 4.6 | -5.0 | 7.51 | 0.79 | VO ventral orbital cortex |
| 0.1 | 3.4 | -6.0 | 7.39 | 0.79 | DP dorsal peduncular |
| -1.5 | 3.2 | -5.4 | 6.93 | 0.76 | VO ventral orbital cortex |
| -0.1 | 3.2 | -6.6 | 7.25 | 0.71 | DTT dorsal tenia tecta |
| -1.1 | 3.8 | -5.2 | 6.18 | 0.64 | MO medial orbital cortex |
| 1.1 | 3.4 | -4.2 | 5.87 | 0.60 | PrL prelimbic cortex |
| 4.1 | -0.4 | -4.4 | 4.46 | 0.37 | Cpu striatum |
| -5.3 | -3.8 | -7.0 | 4.50 | 0.36 | Cpu striatum |
| -4.1 | -11.0 | -6.2 | 4.53 | 0.30 | Crus2 cerebellum |

*Supplementary Table 13. Local maxima of the significant interaction effect of the fMRI BOLD response to somatosensory stimuli between groups and histological data (N=25), where regression slopes between BOLD response and Purkinje cell number are smaller in the VPA600 group than that of the vehicle group. Only local maxima above the FWER P>0.05 threshold (Z>4.37) are shown. Minimum peak distance is set to 0.5mm. Peaks are listed by effect size (% BOLD signal change) in decreasing order.*

| X | Y | Z | Z-score | % signal change | region |
| --- | --- | --- | --- | --- | --- |
| -3.5 | -12.4 | -3.6 | -4.51 | -0.48 | Crus1 cerebellum |
| -2.7 | -12.2 | -5.2 | -4.66 | -0.33 | Crus2 cerebellum |
| -1.9 | -13.6 | -3.4 | -4.46 | -0.30 | Lobule 6 cerebellum |
| -2.1 | -13.2 | -6.8 | -4.43 | -0.28 | Cop Copula pyramis of cerebellum |
| -1.3 | -14.0 | -3.2 | -4.53 | -0.24 | Lobule 6c cerebellum |

*Supplementary Table 14.* Effect of prenatal VPA treatment on average litter size.

Prenatal treatment with VPA did not cause a significant reduction in average litter size in comparison to prenatal saline treatment. The number of male offspring per litter was also not altered by prenatal VPA exposure. Data are presented as mean±SEM.

|  | litter size | male offspring |
| --- | --- | --- |
| Vehicle | 10.2±5.1 | 4.1±2.4 |
| 400 mg/kg VPA | 11±2.3 | 5.1±2.5 |
| 600 mg/kg VPA | 11.5±2.7 | 4.7±2.5 |

# Supplementary Figures


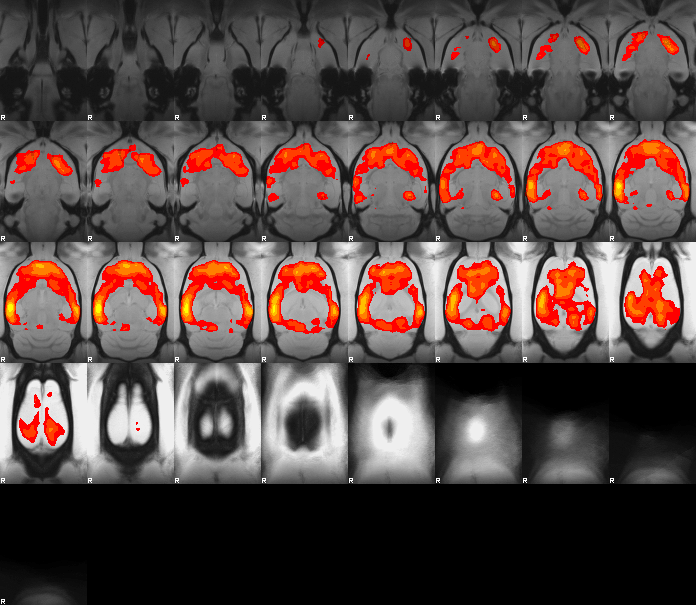


**Supplementary Figure 1. Mean BOLD response to whisker stimulation in the second MRI measurement for the vehicle group.** Z (Gaussianised T/F) statistic images were thresholded using clusters determined by Z>3.1 and a (corrected) cluster significance threshold of P=0.05 (Worsley, 2001)


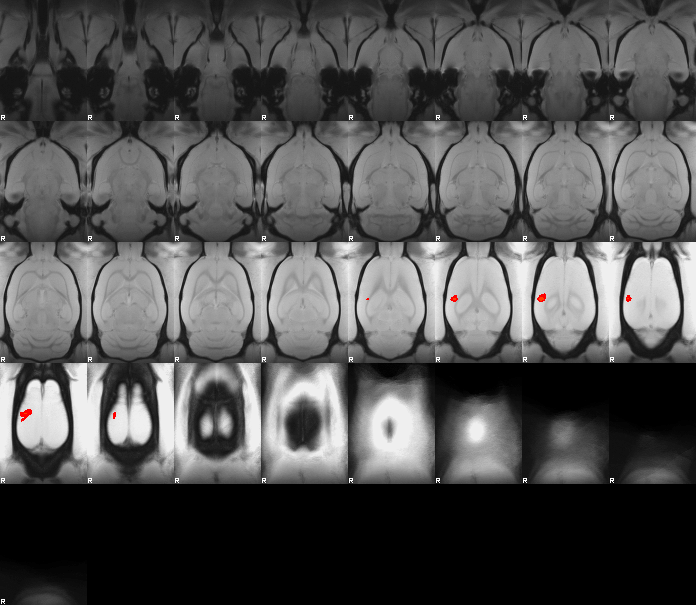


*Supplementary Figure 2. Increased BOLD response to whisker stimulation in the second MRI measurement in the VPA400 group, compared to the vehicle group. Z (Gaussianised T/F) statistic images were thresholded using clusters determined by Z>3.1 and a (corrected) cluster significance threshold of P=0.05 (Worsley, 2001)*


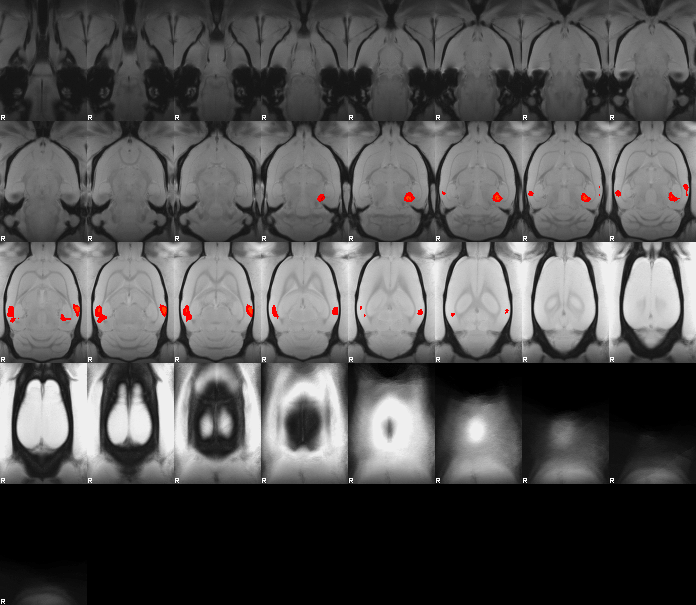


*Supplementary Figure 3. Decreased BOLD response to whisker stimulation in the second MRI measurement in the VPA600 group, compared to the vehicle group. Z (Gaussianised T/F) statistic images were thresholded using clusters determined by Z>3.1 and a (corrected) cluster significance threshold of P=0.05 (Worsley, 2001)*


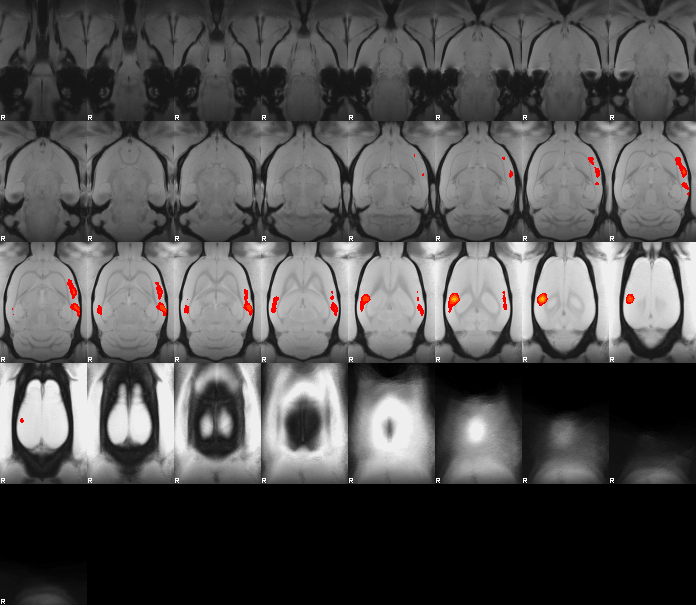


*Supplementary Figure 4. Increased BOLD response to whisker stimulation in the second MRI measurement in the VPA400 group, compared to the VPA600 group. Z (Gaussianised T/F) statistic images were thresholded using clusters determined by Z>3.1 and a (corrected) cluster significance threshold of P=0.05 (Worsley, 2001).*


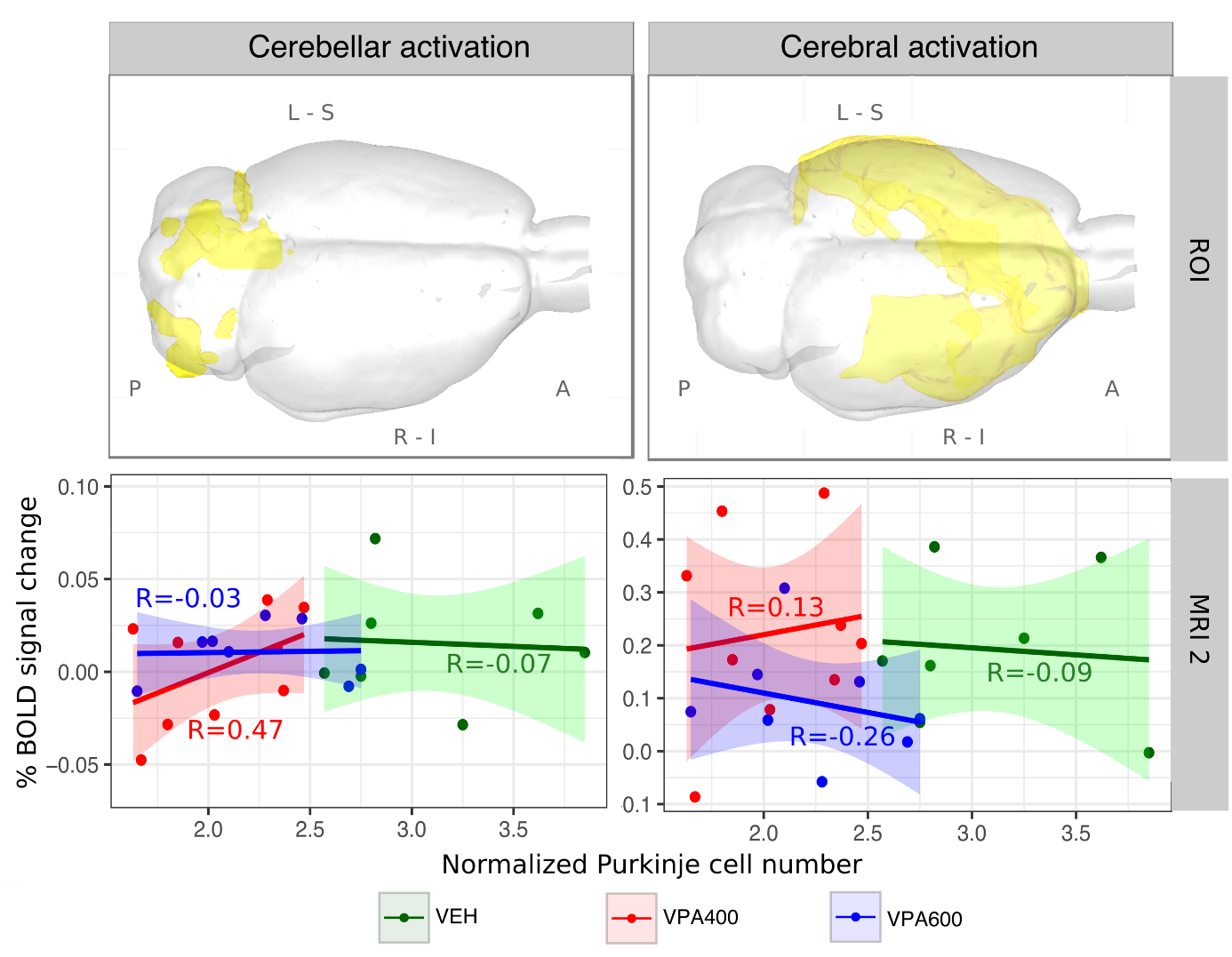


*Supplementary Figure 5. Post-hoc analysis of the “Purkinje cell number x treatment” interaction effect on the BOLD response in ROIs of cerebellar and cerebral BOLD activation in the second MRI measurement.*

*ROIs (visualized in 3D glass-brains) were defined based on the union of the group-wise thresholded (FWER p<0.05) activation maps of the VEH, VPA400 and VPA600 groups and divided into cerebellar (left) and cerebral (right) components. Despite the lower BOLD responses due to anesthesia-related issues with bigger animals, the second MRI measurement confirmed the tendency of positive correlation between BOLD response and Purkinje cell number.*

*Abbreviations: A: anterior, P: posterior, L: left, R: right, S: superior, I: inferior.*


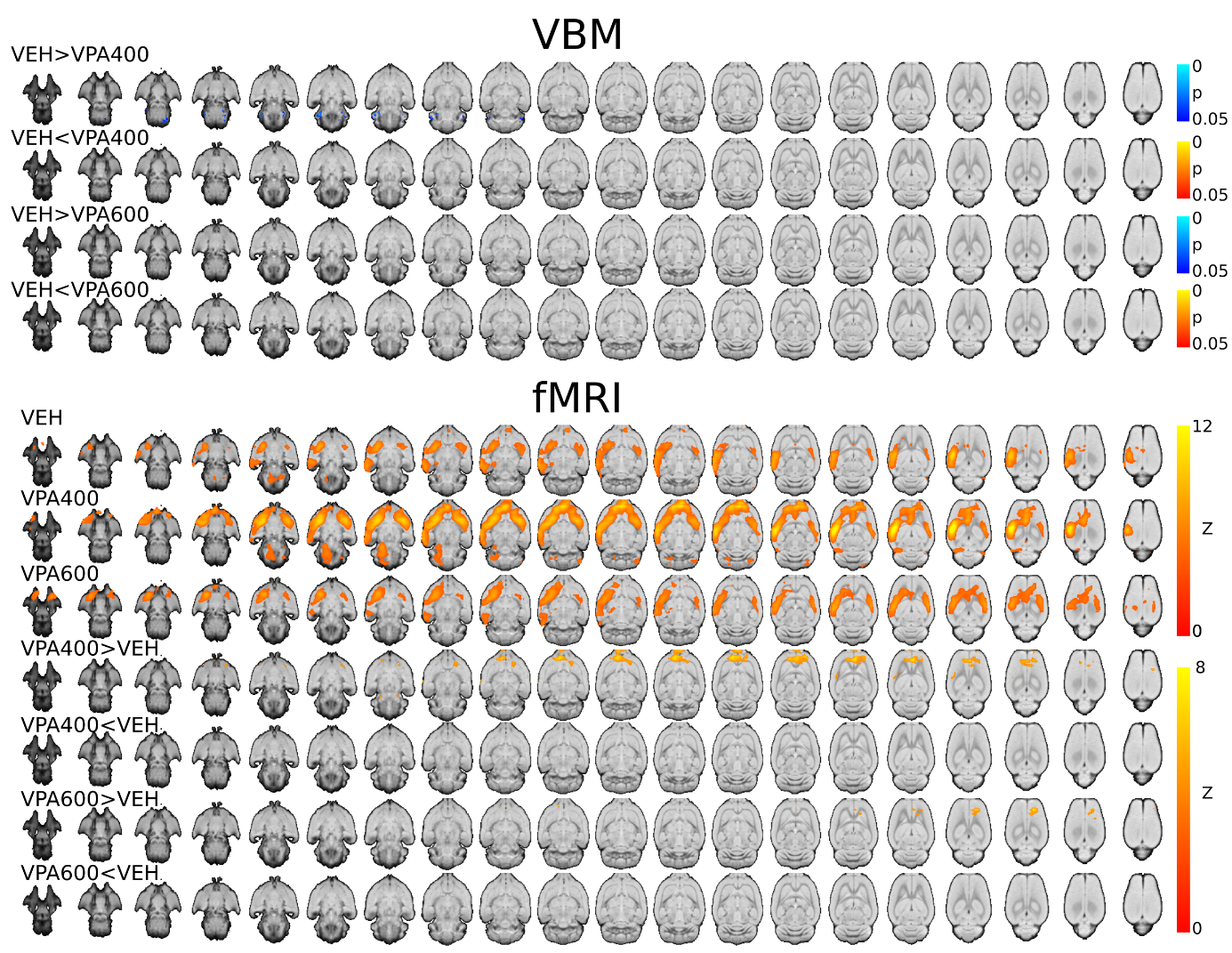


*Supplementary Figure 6: Comprehensive overview of the VBM and fMRI group differences.*

*Twenty slices were taken equidistantly beginning with a slice 9.3 mm ventral from bregma and ending with a slice 1.3 mm from bregma, for all contrasts (distance 0.4 mm). VBM color-bars depicts permutation-based p-values corrected for family-wise error rate (FWER). Blue (red) color-bars denote decreased (increased) VBM gray matter density (no increases were observed). VBM p-value images are thresholded at an FWER-corrected p<0.05 significance threshold. Functional MRI color-bars represent Z-score values of the group-level responses and the group differences. Images are thresholded at an FWER-corrected p<0.05 significance threshold. All statistical parametric images are overlaid on out in-house standard rat template.*


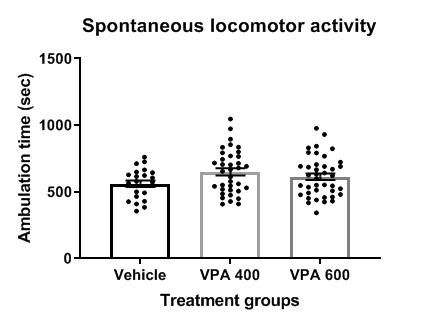


*Supplementary Figure 7. Spontaneous locomotor activity in rats prenatally exposed to VPA (400 and 600mg/kg) and controls at postnatal day 26-28.*

*Spontaneous locomotor activity was measured in male animals at postnatal day 26-28 in a six-channel activity monitor manufactured by Experimetria (Hungary). The apparatus consisted of acrylic cages (48.5cm x 48.5cm x 40cm) equipped with 2 x 30 pairs of photocells along the bottom axis of the cage. Additional arrays of photocells (30 pairs) were placed along two opposite sides of the cage at different heights (6.5, 12, 18 and 23 cm) in order to detect rearing responses. The photocell beam, when broken, signaled a count, which was then recorded by a computer. The signals were processed by a motion analyzing software which determined the spatial position of the animal with 1 Hz sampling frequency, and computed the distance travelled and the time spent by the rats with ambulation, local movement (e.g. grooming), immobility, rearing, etc. Animals were individually placed in the photocell cages and horizontal movements (ambulation time) were determined for one hour. For statistical evaluation one-way ANOVA was used. Data are expressed as mean±SEM (vehicle group n=22, VPA400 group n=36, VPA600 group n=40). VPA treatment did not affect spontaneous locomotor activity (one-way ANOVA F_(2,92)_=2,413; p=0.0952).*


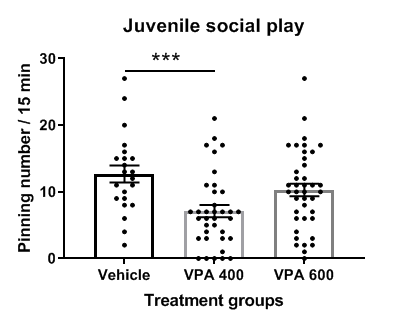


*Supplementary Figure 8. Juvenile social play test in rats prenatally exposed to VPA (400 and 600mg/kg) and controls.*

*Pinning as most characteristic parameter of social play behavior was scored per pair of male animals at postnatal day 33-36. The testing arena of juvenile social play was a plexiglass cage (42 × 42 × 32 cm) with approximately 2 cm of wood shavings covering the floor. Pairs of rats for social interaction were assigned using unfamiliar partners (i.e., not a cage mate or litter mate). Animals in a test pair did not differ more than 10 g in body weight. On postnatal day 34 and 35 each animal was introduced to the testing arena for a period of 5 min individually. On the third day (postnatal day 36), the motivation for play was enhanced by isolating the animals for 4 h before the test. Animals that had been unfamiliar to each other were placed simultaneously into the opposite corners of the previously discovered arena and behavior was recorded for 15 min. Behavioral elements were assessed using the Observer 5.1 software (Noldus Information Technology B.V., The Netherlands). Frequency of pinning as most characteristic parameter of social play behavior was scored per pair of animals and expressed as mean ± SEM (Panksepp, Siviy, & Normansell, 1984; Trezza, Baarendse, & Vanderschuren, 2010). Statistical evaluation was performed by one-way ANOVA followed by Duncan post hoc test, *** p<0.001. Data are expressed as mean±SEM (vehicle group n=22, VPA400 group n=36, VPA600 group n=40). VPA treatment significantly reduced the number of pinnings (one-way ANOVA F_(2,92)_=6.5204; p=0.0022), reduction of pinning behavior reached significance at the dose of 400 mg/kg (p=0.0006 vs. vehicle control) while at the dose of 600 mg/kg, there was no significant effect (p=0.1246 vs vehicle control).*


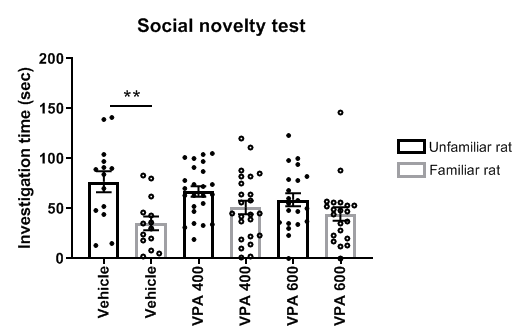


*Supplementary Figure 9. Three chamber social novelty test in rats prenatally exposed to VPA (400 and 600mg/kg) in young adulthood (postnatal day 47-57).*

*In this behavioral assay male offspring (postnatal day 40-47) were used. The paradigm assessed the propensity of the test rat to approach and associate with an unfamiliar, experimentally naive stimulus rat of equal weight and age, and unrelated to the animals of the experimental cohort. The adult social test was performed in a three chambered apparatus. The plexiglass box consisted of three identical chambers with dimensions of 120/40/50 cm (length/width/height). The openings between compartments allowed free exploration of the different chambers. Animals used as “strangers” were Wistar males of the same age and no previous contact with the subject rats. In the experimental protocol, subject rats were initially acclimated for 5 min to the central chamber and then rats were allowed to explore and acclimatize to the all three chambers. In the next session, subject rats could freely move for 10 min in the entire apparatus and investigate either a stranger rat confined behind a perforated separating wall or an empty separated enclosure. In the novelty session, the original stranger rat (familiar rat) remained in its enclosure on one side of the apparatus and a new unfamiliar rat was placed behind a perforated separating wall in the opposite chamber. Measured parameter was time spent with direct exploration of the targets. Following the completion of a test and before the next observation, chambers were thoroughly cleaned first from droppings and urine which was followed by cleaning with 70% ethanol and water; cleaning was finalized with wiping the apparatus dry. Statistical evaluation was performed on the treatment groups separately, using the Student t- test, ** p<0.01. While prenatally vehicle-treated rats were able to discriminate between a novel and a familiar conspecific (ie., spent more time with direct investigation of the novel target animal) (p=0.0013), neither VPA400 (p=0.0546) nor VPA600 (p=0.2614) rats preferred the unknown rat to the familiar one. Data are expressed as mean±SEM (vehicle group n=14, VPA400 group n=24, VPA600 group n=21).*

References

Panksepp, J., Siviy, S., & Normansell, L. (1984). The psychobiology of play: theoretical and methodological perspectives. *Neurosci Biobehav Rev, 8*(4), 465-492.

Trezza, V., Baarendse, P. J., & Vanderschuren, L. J. (2010). The pleasures of play: pharmacological insights into social reward mechanisms. *Trends in pharmacological sciences, 31*(10), 463-469.

Worsley, K. J. (2001). Statistical analysis of activation images. In P. Jezzard, P. M. Matthews, & S. M. Smith (Eds.), *Functional MRI: An Introduction to Methods* (pp. 251-271). Oxford: Oxford Universtiy Press.
